# A retrospective investigation of the platelet-to-lymphocyte ratio as a potential indicator in early rheumatoid arthritis

**DOI:** 10.7717/peerj.21004

**Published:** 2026-03-31

**Authors:** Jingfang Shen, Lianju Li, Lina Leng, Junfeng Li, Xiangzhuo Zhao, Xiaoli Li

**Affiliations:** 1 Department of Rheumatology, Xingtai People’s Hospital, Xingtai, Hebei, China; 2 Department of Neurology, Xingtai People’s Hospital, Xingtai, Hebei, China

**Keywords:** Erythrocyte sedimentation rate, C-reactive protein, Rheumatoid arthritis, Biological markers, Inflammation

## Abstract

**Background:**

The platelet-to-lymphocyte ratio (PLR) has been suggested as a biomarker for inflammation and disease activity in rheumatoid arthritis (RA). However, current findings on its correlation with the Disease Activity Score 28 (DAS28) remain inconsistent. This study aimed to explore the correlation between PLR and DAS28.

**Methods:**

This retrospective study analyzed 433 consecutive inpatients with early RA at Xingtai People’s Hospital between January 2022 and January 2024. Of these, 72.06% were women, with a mean age of 52.52 ± 14.20 years. All patients met the 2010 American College of Rheumatology/European League Against Rheumatism criteria and had symptoms <2 years. To further explore the association between the PLR and the DAS28, multiple regression analyses were conducted, followed by generalized additive models.

**Results:**

After adjusting for potential confounding factors, we found that for every 50-unit increase in PLR, DAS28 based on erythrocyte sedimentation rate (ESR) increases by 0.126 (*P* < 0.001) and DAS28 based on C-reactive protein (CRP) increases by 0.145 (*P* < 0.001). The general trends remained consistent across the PLR tertile groups, from the lowest to the highest. A nonlinear correlation was detected between PLR and DAS28-ESR and DAS28-CRP, with an inflection point for PLR of 254. When PLR was less than 254, it exhibited a significantly positive correlation with DAS28-ESR and DAS28-CRP (*P* < 0.001 for both).

**Conclusion:**

This study revealed a threshold effect: in patients with early RA (ERA), PLR is positively associated with DAS28-ESR and DAS28-CRP only when PLR is below 254. Above that level, the pattern may not hold, illustrating a nonlinear relationship.

## Introduction

Rheumatoid arthritis (RA) is a prevalent chronic inflammatory disease primarily affecting the joints, and may also involve extra-articular manifestations such as rheumatoid nodules, pulmonary complications, vasculitis, and systemic comorbidities. The global prevalence of RA is estimated to be 0.46% (95% confidence interval (CI) 0.39% to 0.54%) (Almutairi et al., 2021). A recent meta-analysis (Mohamed-Ahmed et al., 2024) revealed that the prevalence of RA in China was 354 (210–595) out of 100,000. Poorly controlled RA can lead to joint deformity, disability, reduced quality of life, loss of work capacity, increased risk of comorbidities, and a significant burden on individuals and society (Sokka et al., 2010; Kitas & Gabriel, 2011; Cross et al., 2014). A 2010 global study assessing disability for 291 diseases using years lived with disability ranked RA 42nd (Cross et al., 2014). Consequently, early diagnosis and prompt treatment are imperative to mitigate or prevent subsequent damage. Early RA (ERA) was defined as patients who met the 2010 American College of Rheumatology/European League Against Rheumatism (ACR/EULAR) classification criteria for RA (Kay & Upchurch, 2012) for RA on a short-term basis, usually < 1 year or the duration of RA < 2 years (Young et al., 2000; O’Neil, Alpízar-Rodríguez & Deane, 2024). To ensure optimal management of RA, early treatment and targeted therapy should be pursued (Salomon-Escoto & Kay, 2019).

Accurate disease assessment is paramount for the implementation of standardized treatment. The erythrocyte sedimentation rate (ESR) and C-reactive protein (CRP), as our commonly used clinical assessment tools for RA disease activity, have certain limitations, such as the study found that about half of the patients with normal CRP had inflammatory activity on synovial biopsy (Orr et al., 2018) and the North American Federation of Rheumatology Investigators database found that among 9,135 patients with active RA, 58% of the patients had normal ESR and CRP (Kay et al., 2014). While the Disease Activity Score 28 (DAS28) is a commonly used composite tool for assessing RA in clinical practice and research (Son et al., 2016), its application is often cumbersome and somewhat subjective.

Thus, there is an urgent need for novel biomarkers that can better evaluate disease activity and enable more precise stratification of RA. Beyond RA, hematological ratios such as the neutrophil-to-lymphocyte ratio (NLR) and platelet-to-lymphocyte ratio (PLR) have been established as cost-effective inflammatory biomarkers in related diseases. Their significant correlation with disease activity scores has been demonstrated in systemic lupus erythematosus (Soliman et al., 2020; Abdalhadi et al., 2023) and ankylosing spondylitis (Zeb et al., 2019). The numbers of lymphocytes (LYM), platelets (PLT), and neutrophils (NEUT), or their ratio in peripheral blood, were estimated or predicted to play a role in systemic inflammatory response in RA management, such as the PLR, as a cellular marker derived from routine hematological parameters has been used to propose as a predictive marker for RA and RA-associated interstitial lung disease (RA-ILD) (Chen et al., 2019). Cheng et al. (2024) found that in RA patients with normal ESR and CRP, PLR is positively associated with ultrasound-detected synovitis and bone erosion in RA patients. A study on RA reported that the PLR is positively correlated with ESR, CRP, and DAS28-ESR (Meng et al., 2024). A study in Korea found that baseline PLR was associated with DAS28-ESR (*p* < 0.05) in patients with RA receiving Janus kinase inhibitor therapy. Moreover, changes in PLR during treatment were also correlated with DAS28-ESR, although the reduction in PLR did not reach statistical significance (*p* = 0.056) (Choe & Kim, 2022). These studies suggest that PLR may be a biomarker for assessing disease activity in patients with RA.

However, existing studies have reported inconsistent findings regarding the association between PLR and DAS28 (Chen et al., 2019; Boulos et al., 2019). This study aimed to investigate this correlation and clarify whether PLR can identify disease activity in patients with ERA.

## Materials and Methods

### Study design and population

**Figure 1:**
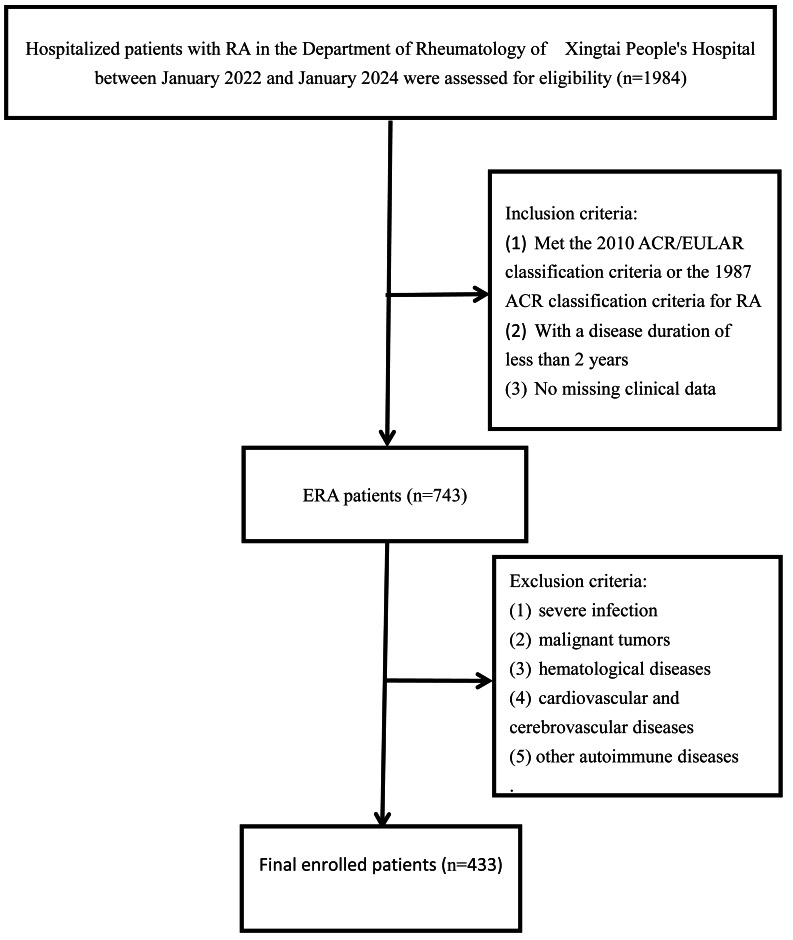
Flow chart on selection of patients. RA, Rheumatoid arthritis; ERA, Early rheumatoid arthritis; ACR, American College of Rheumatology; EULAR, European League Against Rheumatism.

We performed a retrospective study by collecting data from 433 consecutive patients with ERA, who were hospitalized at Xingtai People’s Hospital from January 2022 to January 2024 (Fig. 1). The term ‘hospitalized’ in this context specifically refers to admission for the purpose of systematic evaluation and initiation of intensive therapy, prompted by high disease activity (*e.g.*, significant joint involvement and markedly elevated inflammatory markers). All the patients met the 2010 ACR/EULAR classification criteria for RA (Kay & Upchurch, 2012), with a disease duration of less than 2 years. The definitive diagnosis was established through systematic collection and cross-verification of complete clinical, laboratory, and imaging data obtained after admission. Screening for comorbidities was based on comprehensive medical history, thorough laboratory testing, and necessary ancillary examinations conducted following hospitalization.

Exclusion criteria included severe infection, malignant tumors, hematological diseases, cardiovascular and cerebrovascular diseases, and other autoimmune diseases. The study was approved by the Ethics Committee of Xingtai People’s Hospital (Approval No. 2025[031]) on February 13, 2025, and due to the retrospective nature of this study, the requirement for informed consent was waived. Besides, this study was conducted in compliance with the Strengthening the Reporting of Observational Studies in Epidemiology (STROBE) statement and the Declaration of Helsinki.

### Data collection and definitions

Baseline demographics, details of current therapy, smoking and drinking status, and symptom-to-admission interval (SAI) were retrieved. The following parameters from the full blood examination were collected: leukocyte (WBC, 10^9^/L), NEUT (10^9^/L), LYM (10^9^/L), monocyte (MONO, 10^9^/L) and red blood cell counts (RBC, 10^12^/L), hemoglobin (HGB, g/L), PLT (10^9^/L), and platelet distribution width (PDW). Body Mass Index (BMI) was calculated as weight by height squared (kg/m^2^). The PLR was calculated by dividing the PLT count by the LYM count (PLR = PLT / LYM). Disease activity was assessed using DAS28-ESR and DAS28-CRP. All component data were systematically extracted from patients’ electronic hospital records. This included: (1) the 28-joint tender joint count (TJC28) and swollen joint count (SJC28), obtained from detailed physical examination notes; (2) the patient’s global assessment of disease activity (GH), recorded on a 0–100 mm visual analog scale by the attending physician at admission; and (3) laboratory measurements of ESR and CRP from blood samples collected at admission per standard protocols.

The scores were calculated using the following standard formulas (Sengul et al., 2015):

DAS28-ESR = 0.56 ×√(TJC28) + 0.28 × √(SJC28) + 0.70 × ln(ESR) + 0.014 × GH

DAS28-CRP = 0.56 ×√(TJC28) + 0.28 × √(SJC28) + 0.36 × ln(CRP + 1) + 0.014 × GH + 0.96 (note: A value of 1 was added to CRP prior to the natural logarithm to handle cases where CRP equaled zero.)

### Statistical analysis

Categorical variables were presented as percentages and compared using the chi-square test. Continuous variables were described as mean ± standard deviation (SD) for parametric data or median and interquartile range (IQR) for non-parametric data, with group comparisons using one-way analysis of variance (ANOVA) or Kruskal–Wallis H test. Three logistic regression models were fitted: the original Model (unadjusted PLR effect); Model 1 (PLR plus sex, age, SAI); and Model 2 (adds MONO, NEUT, HGB, the use of glucocorticoid, conventional synthetic disease-modifying antirheumatic drugs (csDMARDs), and biologic disease-modifying antirheumatic drugs (bDMARDs) to Model 1 covariates). Because PLR, as a continuous variable, does not have a clinical threshold, we artificially divided it into three groups. We not only present the results of its constant variable, but also use it as a categorical variable to measure its correlation with DAS28-ESR and DAS28-CRP. This study employed a stepwise statistical strategy to analyze the relationship between PLR and disease activity. First, a generalized additive model (GAM) with nonparametric smoothing functions was used to explore potential nonlinear associations. The complexity of the smooth terms was controlled using thin-plate regression splines. The significance of non-linearity was assessed by the approximate *F*-test of the smooth term, with the effective degrees of freedom (edf) indicating the shape of the association. The statistically significant result (*p* < 0.05) supported proceeding to a threshold analysis. Subsequently, a maximum likelihood-based two-piecewise linear regression model was applied to automatically identify the inflection point and to separately estimate the slopes, along with their 95% confidence intervals, on either side of the inflection point. The statistical significance of the inflection point was verified using a likelihood ratio test. All analyses were performed using R (version 4.5.2; R Core Team, 2026) and EmpowerStats (http://www.empowerstats.com/). A two-sided *p* < 0.05 was considered statistically significant.

## Results

### Characteristics of the patients

Based on the inclusion and exclusion criteria, the final sample size included 433 participants. Table 1 presents the characteristics of the overall study subjects stratified by tertiles of PLR. The study population consisted of 312 (72.06%) females, with a mean age of 52.52 ± 14.20 years. The mean values of DAS28-ESR and DAS28-CRP were 5.36 ± 1.12 and 4.72 ± 1.10. There were significant differences in drinking, ESR, CRP, LYM, RBC, HGB, PLT, PDW, DAS28-ESR, DAS28-CRP, the types of csDMARDs used, and bDMARDs among the three groups. Subjects with higher PLR had higher ESR, CRP, PLT, DAS28-ESR, and DAS28-CRP compared to those with lower PLR (*P* < 0.001). The proportion of patients utilizing csDMARDs and bDMARDs was higher in the higher PLR tertile than in the lower tertiles (both *P* = 0.002). Moreover, subjects in the lower PLR group had higher LYM, HGB, and PDW compared to those in the higher PLR groups (*P* < 0.001). However, there were no significant differences in other variables among the three groups.

**Table 1:** Baseline characteristics of the included participants.

		Tertiles of PLR			
Characteristics	Total (44.42–720.51)	T1 (44.42–148.77)	T2 (149.18–217.46)	T3 (217.50–720.51)	*P* value
N	433	144	144	145	
Sex (female), n (%)	312 (72.06%)	100 (69.44%)	105 (72.92%)	107 (73.79%)	0.685
Age (year), mean ± SD	52.52 (14.20)	52.21 (14.14)	51.94 (14.09)	53.39 (14.41)	0.403
BMI (kg/m^2^), mean ± SD	23.95 (3.46)	24.35 (3.67)	24.10 (3.42)	23.39 (3.24)	0.072
SAI (month), median (IQR)	4.00 (2.00–10.00)	6.00 (2.00–10.50)	4.00 (2.00–10.00)	4.00 (2.00–12.00)	0.556
Smoking, n (%)	14 (3.23%)	5 (3.47%)	4 (2.78%)	5 (3.45%)	0.574
Drinking, n (%)	6 (1.39%)	2 (1.39%)	4 (2.78%)	0 (0.00%)	0.020
ESR (mm/h), median (IQR)	57.00 (32.50–79.00)	42.00 (23.00–63.00)	54.00 (31.00–71.00)	76.00 (57.50–90.25)	<0.001
CRP (mg/L), median (IQR)	25.12 (8.59–53.10)	15.07 (3.15–28.79)	19.67 (7.99–43.04)	49.30 (27.53–68.12)	<0.001
WBC (×10^9^/L), mean ± SD	6.70 (2.22)	6.90 (1.91)	6.64 (2.39)	6.56 (2.32)	0.096
LYM (×10^9^/L), mean ± SD	1.64 (0.63)	2.11 (0.61)	1.62 (0.49)	1.20 (0.38)	<0.001
MONO (×10^9^/L), mean ± SD	0.51 (0.20)	0.51 (0.21)	0.49 (0.19)	0.51 (0.21)	0.860
NEU (×10^9^/L), mean ± SD	4.39 (1.86)	4.12 (1.46)	4.34 (1.96)	4.70 (2.06)	0.085
RBC (×10^12^/L), mean ± SD	4.05 ± 1.66	4.10 (0.57)	3.94 (0.40)	4.12 (2.79)	<0.001
HGB (g/L), mean ± SD	116.03 (16.19)	123.34 (15.21)	115.86 (14.54)	108.94 (15.58)	<0.001
PLT (×10^9^/L), mean ± SD	295.08 (95.81)	241.83 (71.25)	288.71 (83.69)	354.28 (95.58)	<0.001
PDW, mean ± SD	14.85 (2.93)	15.10 (2.87)	14.91 (2.87)	14.54 (3.04)	<0.001
DAS28-ESR, mean ± SD	5.36 (1.12)	4.80 (1.16)	5.35 (1.02)	5.91 (0.90)	<0.001
DAS28-CRP, mean ± SD	4.72 (1.10)	4.19 (1.10)	4.69 (0.99)	5.26 (0.93)	<0.001
Glucocorticoids, %	37 (8.55%)	12 (8.33%)	10 (6.94%)	15 (10.34%)	0.582
Types of csDMARDs					0.002
0	72 (16.63%)	35 (24.31%)	21 (14.58%)	16 (11.03%)	
1	307 (70.90%)	101 (70.14%)	102 (70.83%)	104 (71.72%)	
2	54 (12.47%)	8 (5.56%)	21 (14.58%)	25 (17.24%)	
bDMARDs,%	99 (22.92%)	21 (14.58%)	32 (22.38%)	46 (31.72%)	0.002

**Notes.**

Abbreviations PLR platelet-to-lymphocyte ratio BMI body mass index SAI symptom-to-admission interval WBC leukocyte LYM lymphocyte MONO monocyte NEUT neutrophil RBC red blood cell HGB hemoglobin PLT platelet PDW platelet distribution width ESR erythrocyte sedimentation CRP C-reactive protein DAS28 disease activity score in 28 joints csDMARDs conventional synthetic disease-modifying antirheumatic drugs bDMARDs biological disease-modifying antirheumatic drugs

As illustrated in Table 2, univariate analysis revealed that DAS28-ESR was positively associated with age, ESR, CRP, PLT, PLR (*P* < 0.001 for all), MONO (*P* = 0.029), NEUT (*P* = 0.001), as well as the use of glucocorticoids (*P* = 0.004), csDMARDs (1 *versus* 0 types: *P* = 0.031; 2 *versus* 0 types: *P* = 0.003), and bDMARDs (*P* = 0.001). Conversely, DAS28-ESR exhibited a negative correlation with LYM (*P* = 0.001) and HGB (*P* < 0.001). Furthermore, the DAS28-CRP score demonstrated significant associations with various parameters. Specifically, it showed a positive correlation with age, ESR, CRP, PLR, neutrophil count, PLT (*P* < 0.001 for all), MONO and WBC (both *P* = 0.001), the use of csDMARDs (1 *versus* 0 types: *P* = 0.080; 2 *versus* 0 types: *P* = 0.026), bDMARDs (*P* = 0.001), and glucocorticoids (*P* = 0.001); however, it exhibited a negative correlation with LYM (*P* = 0.001) and HGB (*P* < 0.001).

**Table 2:** Univariable analysis of DAS28-ESR and DAS28-CRP with PLR and confounders.

Characteristics	DAS28-ESR β (95% CI)	DAS28-ESR *P* value	DAS28-CRP β (95% CI)	DAS28-CRP *P* value
Female	Ref	Ref	Ref	
Male	−0.077 (−0.313, 0.159)	0.522	0.194 (−0.036, 0.424)	0.100
Age (year)	0.025 (0.018, 0.032)	<0.001	0.026 (0.019, 0.033)	<0.001
SAI (month)	−0.009 (−0.026, 0.008)	0.283	−0.012 (−0.028, 0.005)	0.162
BMI (kg/m^2^)	−0.017 (−0.048, 0.013)	0.270	−0.012 (−0.042, 0.018)	0.444
ESR (mm/H)	0.028 (0.026, 0.031)	<0.001	0.023 (0.020, 0.026)	<0.001
CRP (mg/L)	0.017 (0.014, 0.019)	<0.001	0.020 (0.018, 0.022)	<0.001
WBC (×10^9^/L)	0.047 (−0.001, 0.095)	0.053	0.090 (0.044, 0.136)	0.001
LYM (×10^9^/L)	−0.347 (−0.513, −0.181)	0.001	−0.287 (−0.451, −0.124)	0.001
NEUT (×10^9^/L)	0.097 (0.040, 0.153)	0.001	0.146 (0.092, 0.200)	<0.001
MONO (×10^9^/L)	0.577 (0.060, 1.093)	0.029	0.996 (0.497, 1.495)	0.001
RBC (×10^9^/L)	−0.025 (−0.088, 0.039)	0.450	−0.004 (−0.067, 0.058)	0.894
HGB (g/L)	−0.027 (−0.033, −0.021)	<0.001	−0.020 (−0.026, −0.014)	<0.001
PLT (×10^9^/L)	0.004 (0.003, 0.005)	<0.001	0.004 (0.003, 0.005)	<0.001
PDW	−0.008 (−0.044, 0.029)	0.682	−0.005 (−0.041, 0.030)	0.772
PLR	0.005 (0.004, 0.006)	<0.001	0.005 (0.004, 0.006)	<0.001
Glucocorticoids, %	0.547 (0.172, 0.922)	0.004	0.610 (0.244, 0.976)	0.001
types of csDMARDs				
0	Ref		Ref	
1	0.315 (0.029, 0.600)	0.031	0.251 (−0.030, 0.532)	0.080
2	0.607 (0.214, 0.999)	0.003	0.440 (0.054, 0.826)	0.026
bDMARDs, %	0.457 (0.208, 0.705)	0.001	0.405 (0.162, 0.649)	0.001

**Notes.**

Abbreviations SAI symptom-to-admission interval BMI body mass index WBC leukocyte LYM lymphocyte MONO monocyte NEUT neutrophil RBC red blood cells HGB hemoglobin PLT platelet PDW platelet distribution width ESR erythrocyte sedimentation CRP C-reactive protein PLR Platelet to Lymphocyte Ratio csDMARDs conventional synthetic disease-modifying antirheumatic drugs bDMARDs biological disease-modifying antirheumatic drugs

### Association between PLR and DAS28-ESR and DAS28-CRP

Multiple linear regression models were employed to determine PLR’s association with DAS28-ESR and DAS28-CRP. Table 3 demonstrates that for every 50-unit increase in PLR, DAS28-ESR increases by 0.238 (*P* < 0.001) and DAS28-CRP increases by 0.238 (*P* < 0.001) in the unadjusted model, which does not control for confounders. Model I, which adjusts for basic demographic variables, shows that a 50-unit increase in PLR is associated with DAS28-ESR β = 0.218 (*P* < 0.001) and DAS28-CRP β = 0.222 (*P* < 0.001). In Model II, which further adjusts for clinical variables, consistent positive correlations were observed with a 50-unit increase in PLR and DAS28-ESR (β = 0.126, 95% CI [0.076–0.177], *P* < 0.001) and DAS28-CRP (β = 0.145, 95% CI [0.095–0.194], *P* < 0.001).

**Table 3:** Multivariable linear regression analysis of the association between PLR and DAS28-ESR and DAS28-CRP.

Outcome	Predictor	Crude model β (95% CI)	*P* value	Adjusted Model I β (95% CI)	*P* value	Adjusted Model II β (95% CI)	*P* value
**DAS28-ESR**		
	PLR (per unit)	0.005 (0.004, 0.006)	<0.001	0.004 (0.003, 0.005)	<0.001	0.003 (0.002, 0.004)	<0.001
	PLR (per 50 units)	0.238 (0.186, 0.290)	<0.001	0.218 (0.168, 0.268)	<0.001	0.126 (0.076, 0.177)	<0.001
	**PLR (Tertiles)**						
	T1	Ref		Ref		Ref	
	T2	0.551 (0.313, 0.789)	<0.001	0.550 (0.326, 0.775)	<0.001	0.347 (0.130, 0.564)	0.002
	T3	1.107 (0.869, 1.344)	<0.001	1.069 (0.845, 1.293)	<0.001	0.649 (0.416, 0.882)	<0.001
	*P* for trend	<0.001		<0.001		<0.001	
**DAS28-CRP**		
	PLR (per unit)	0.005 (0.004, 0.006)	<0.001	0.004 (0.003, 0.005)	<0.001	0.003 (0.002, 0.004)	<0.001
	PLR (per 50 units)	0.238 (0.187, 0.288)	<0.001	0.222 (0.173, 0.270)	<0.001	0.145 (0.095, 0.194)	<0.001
	**PLR (Tertiles)**						
	T1	Ref		Ref		Ref	
	T2	0.498 (0.265, 0.732)	<0.001	0.505 (0.286, 0.724)	<0.001	0.352 (0.138, 0.566)	0.001
	T3	1.070 (0.837, 1.302)	<0.001	1.045 (0.826, 1.263)	<0.001	0.703 (0.473, 0.933)	<0.001
	*P* for trend	<0.001		<0.001		<0.001	

**Notes.**

**Crude Model: **Unadjusted.

**Adjusted Model I: **Adjusted for sex, age, and SAI.

**Adjusted Model II: **Additionally adjusted for monocyte count, neutrophil count, hemoglobin, and the use of glucocorticoids, conventional synthetic disease-modifying antirheumatic drugs (csDMARDs), and biological disease-modifying antirheumatic drugs (bDMARDs).

Abbreviations PLR platelet-to-lymphocyte ratio DAS28-ESR Disease Activity Score in 28 joints using erythrocyte sedimentation rate DAS28-CRP Disease Activity Score in 28 joints using C-reactive protein SAI symptom-to-admission interval csDMARDs conventional synthetic disease-modifying antirheumatic drugs bDMARDs biological disease-modifying antirheumatic drugs

### Nonlinear relationship

**Figure 2:**
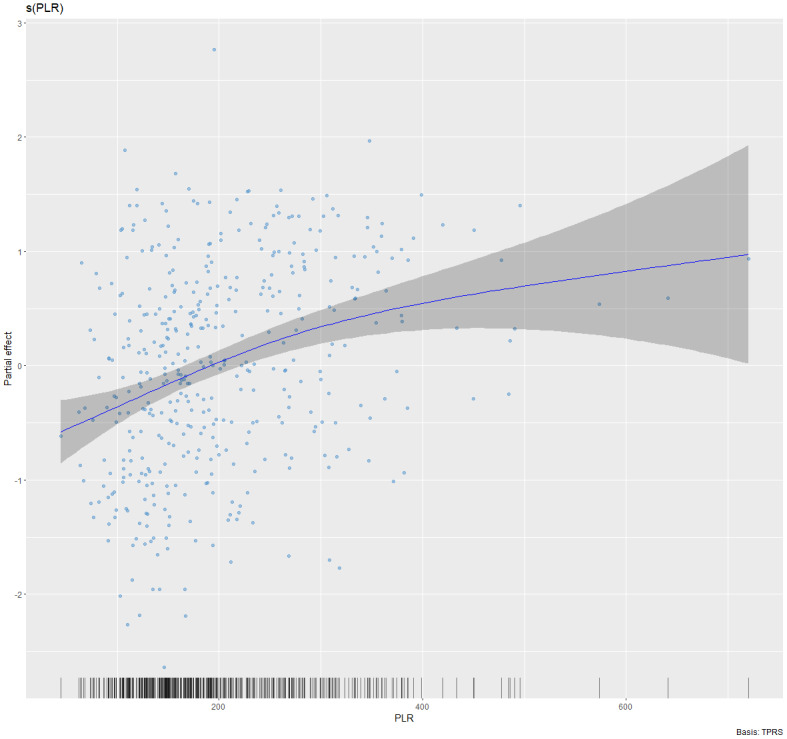
Non-linear association between PLR and DAS28-ESR in rheumatoid arthritis. The fitted smooth curve (solid line) and 95% confidence interval (shaded area) are derived from a Generalized Additive Model (GAM), adjusted for sex, age, symptom-to-admission interval, monocyte count, neutrophil count, hemoglobin level, use of glucocorticoids, bDMARDs, and types of csDMARDs (e*df* = 2.174, *p* < 0.001). An edf > 2 indicates a non-linear relationship.

**Figure 3:**
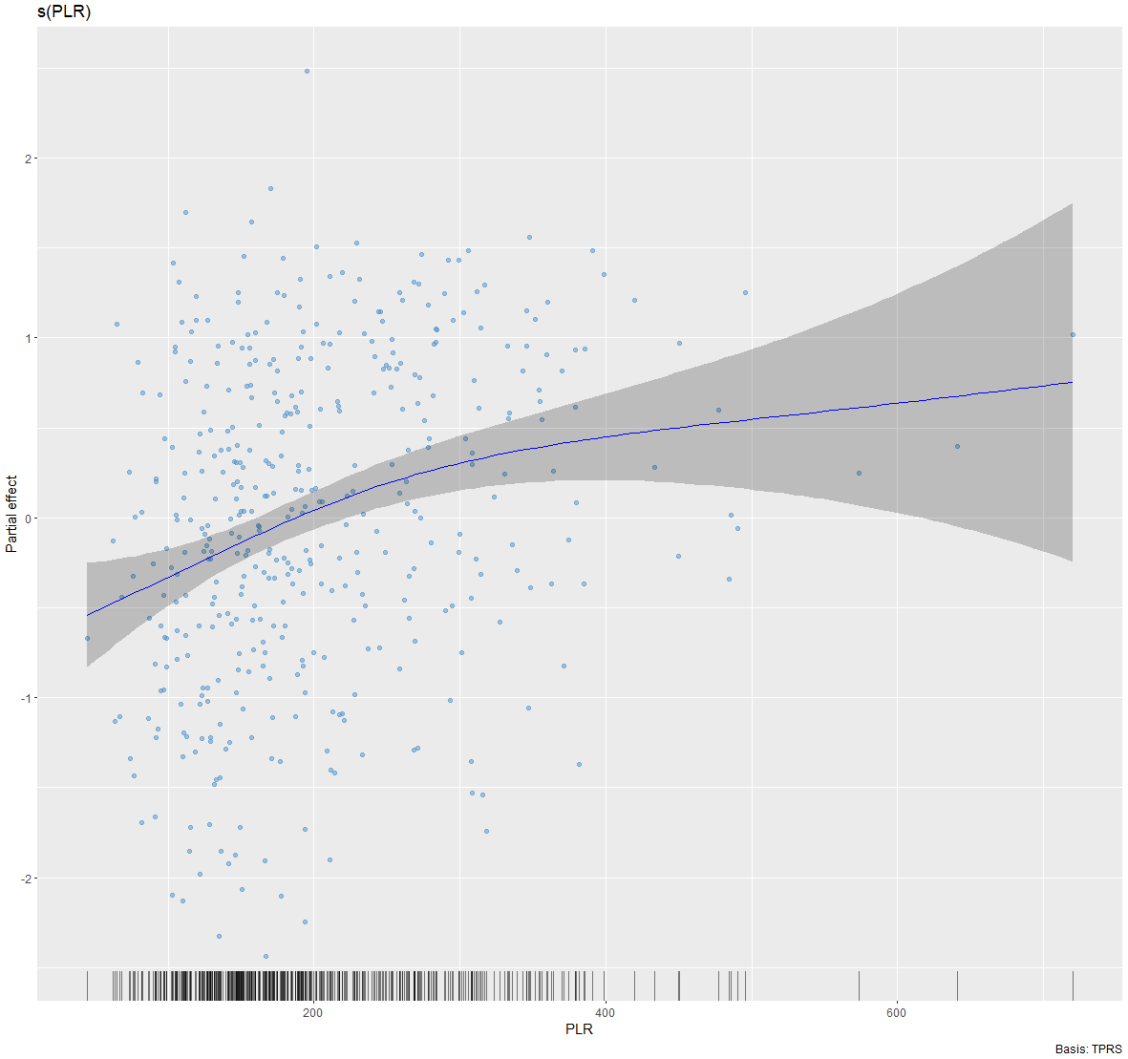
Non-linear association between PLR and DAS28-CRP in rheumatoid arthritis. The fitted smooth curve (solid line) and 95% confidence interval (shaded area) are derived from a Generalized Additive Model (GAM), adjusted for sex, age, symptom-to-admission interval, monocyte count, neutrophil count, hemoglobin level, use of glucocorticoids, bDMARDs, and types of csDMARDs (e*df* = 2.059, *p* < 0.001). An edf > 2 indicates a non-linear relationship.

Given that both DAS28 and PLR are continuous variables, it was essential to investigate the potential presence of a nonlinear association. In the fully adjusted GAM model controlling for sex, age, SAI, MONO, NEUT, HGB, the use of glucocorticoids and bDMARDs, types of csDMARDs, the analysis revealed a significant non-linear association. The PLR showed a significant non-linear association with both disease activity indices. Specifically, the smooth term for PLR and DAS28-ESR was statistically significant (effective degrees of freedom (edf) = 2.174, *p* < 0.001), indicating a non-linear relationship. Similarly, the association between PLR and DAS28-CRP was also non-linear and significant (e*df* = 2.059, *p* < 0.001). The partial effect plots reveal that the relationship between PLR and disease activity follows a monotonic increasing trend until a plateau (see Figs. 2 and 3). To simplify clinical interpretation, we applied a common threshold of PLR = 254 for both disease activity indices, as their independently estimated inflection points were closely aligned (253.35 for DAS28-ESR and 254.37 for DAS28-CRP). Using this unified threshold, a piecewise linear regression model revealed a significant threshold effect in the association with PLR after adjusting for covariates (likelihood ratio test *P* = 0.007 for DAS28-ESR and *P* = 0.015 for DAS28-CRP) (Table 4). When PLR was less than 254, both DAS28-ESR and DAS28-CRP exhibited a significantly positive correlation with PLR (β = 0.005, 95% CI [0.003–0.006] and β = 0.005, 95% CI [0.003–0.007], *P* < 0.001). However, when PLR exceeded 254, DAS28-ESR and DAS28-CRP were not significantly correlated with PLR (β = 0.000, 95% CI [−0.002–0.002], *P* = 0.717 and β = 0.001, 95% CI [−0.001–0.003], *P* = 0.331).

**Table 4:** Threshold effect analysis of platelet-to-lymphocyte ratio (PLR) on disease activity scores using two-piecewise linear regression.

	DAS28-ESR β (95% CI)	*P* value	DAS28-CRP β (95% CI)	*P* value
Fitting model by standard linear regression	0.003 (0.002, 0.004)	<0.001	0.003 (0.002, 0.004)	<0.001
Fitting model by two-piecewise linear regression			
Inflection point of PLR	253.35		254.37	
<254	0.005 (0.003, 0.006)	<0.001	0.005 (0.003, 0.007)	<0.001
≥254	0.000 (−0.002, 0.002)	0.717	0.001 (−0.001, 0.003)	0.331
*P* for log likelihood ratio test	0.007		0.015	

**Notes.**

The two-piecewise linear regression identified optimal inflection points at PLR = 253.35 for DAS28-ESR and PLR = 254.37 for DAS28-CRP. For model simplification and clinical interpretability, a common threshold of PLR = 254 was used for the stratified analysis presented below. All models were adjusted for sex, age, SAI, monocyte count, neutrophil count, hemoglobin, and the use of glucocorticoids, csDMARDs, and bDMARDs.

Abbreviations PLR platelet-to-lymphocyte ratio DAS28-ESR Disease Activity Score in 28 joints using erythrocyte sedimentation rate DAS28-CRP Disease Activity Score in 28 joints using C-reactive protein SAI symptom-to-admission interval csDMARDs conventional synthetic disease-modifying antirheumatic drugs bDMARDs biological disease-modifying antirheumatic drugs

## Discussion

PLR is associated with various inflammatory and immunologic diseases, such as RA, ankylosing spondylitis (AS) (Wang et al., 2022), and systemic lupus erythematosus (SLE) (Lee & Song, 2024). In this cross-sectional study, we analyzed the correlation between PLR and DAS28 in patients with ERA, and found that the correlation remained significant after adjusting for multiple confounders. In addition, we found a nonlinear relationship between PLR and DAS28. To our knowledge, this was the first study to discover a threshold effect: in patients with ERA, PLR is positively associated with disease activity (DAS28 scores), only when PLR is below 254. Above that level, the pattern may not hold, illustrating a nonlinear relationship.

This research indicated that the PLR exhibited a positive correlation with both DAS28-ESR and DAS28-CRP, broadly aligning with findings from previous studies (Xu et al., 2022). A multicenter retrospective study (Xu et al., 2022) on RA performed both univariate and multivariate regression analyses, revealing a statistically significant positive association between PLR and DAS28-ESR as well as DAS28-CRP. The study conducted in China reported a significant correlation between PLR and DAS28 by multiple linear regression analysis in patients with RA and RA-ILD (Chen et al., 2019) (β = 0.374, *p* < 0.001, 95% CI [0.003–0.005]), after adjustment for age, sex, BMI, NLR, lymphocyte-monocyte ratio (LMR), and PLT, LYM, NEUT, and MONO. Due to differences in enrolled patients, we obtained different partial regression coefficients, all of which indicated a positive correlation between PLR and DAS28. A Turkish study using a single-factor analysis method found a correlation between PLR and DAS28 (*r* = 0.352, *P* ≤ 0.0001) in RA patients (Uslu et al., 2015). A study investigating the correlation between systemic inflammatory hematological markers and ultrasonographic disease activity parameters in patients with RA revealed that PLR was positively correlated not only with multiple ultrasonographic markers of disease activity but also with the following clinical parameters: DAS28, TJC, SJC, patient global assessment (PGA), and morning stiffness duration (Targońska-Stępniak et al., 2020).

However, Cheng et al. (2024) found no significant correlation between PLR and DAS28-ESR, DAS28-CRP in RA patients with normal acute-phase reactant (APR) levels. The underlying mechanism behind this discrepancy is unknown, probably because the latter chose patients with normal APR levels. In a study conducted in South Korea, patients with RA were categorized into three subgroups based on DAS28-ESR and DAS28-CRP: remission, low disease activity, and moderate to high disease activity. The PLR showed an increasing trend with worsening disease activity. However, while the difference in PLR was statistically significant in the DAS28-ESR-based classification (*p* = 0.021), it was not statistically significant in the DAS28-CRP-based classification (*p* > 0.05) (Choe, Lee & Kim, 2023). The lack of adjustment for potential confounders in the univariate analysis may, in part, explain why a significant correlation between PLR and DAS28-CRP was not observed. In contrast, the multiple linear regression model employed in our study, after controlling for confounding variables, effectively demonstrated robust positive correlations of PLR with both DAS28-CRP and DAS28-ESR.

In recent years, there has been a gradual growth in the literature on the role of PLR in RA. However, the present study analyzed the relationship between PLR and RA disease activity more thoroughly than previous studies. Previous studies have found that patients with elderly onset RA (EORA) (onset > 60 years) have a lower PLR than those with young onset RA (YORA) (onset at a younger age) (Targońska-Stępniak et al., 2021), illustrating the influence of age and medical history on PLR. MONO plays a role in the progression of RA (Salnikova et al., 2024). In addition, NEUT has been implicated in joint destruction in inflammatory arthritis (Boulos et al., 2019). Given their role in the disease activity or pathogenesis of RA, these factors could influence the PLR and thus interfere with our assessment of RA. To ensure the accuracy of our results, we considered and corrected these confounding factors and developed three models to separate the effects of SAI, age, MONO, and NEUT. The stability we discovered indicates the relationship between PLR and RA was considerable.

Additionally, smooth curve fitting demonstrated a nonlinear association between PLR and DAS28. Meanwhile, a recently published study on the relationship between PLR and mortality in critically ill patients with RA found that PLR is a significant prognostic indicator for critically ill RA patients, demonstrating a U-shaped association with 28-day and 90-day mortality (Yuan et al., 2025). Another study also indicated a nonlinear relationship between SII, PLR, and all-cause mortality in RA patients (Liu et al., 2025). This study reveals that a specific range of PLR may be beneficial in evaluating RA. To clarify the clinical significance of the correlation between PLR and DAS28-ESR/CRP identified in this study, we used the DAS28 reduction threshold of ≥0.6 from the EULAR response criteria as a reference for quantitative assessment (Fransen & Van Riel, 2009). Model analysis revealed that when PLR is below 254, a decrease of approximately 126 and 130 units is associated with a clinically meaningful improvement in DAS28-ESR and DAS28-CRP. It should be emphasized that this finding is based on correlational extrapolation from cross-sectional data and does not establish causality. Future interventional studies are needed to further validate the actual impact of PLR on disease activity.

Increased PLT counts and decreased LYM counts in systemic inflammatory disorders result in a rise in PLR (Liang et al., 2021). The elevated PLR levels in ERA may be due to the chronic inflammatory response in ERA. The pathogenesis of RA is a combination of innate and adaptive immunity (Das et al., 2019), with PLT being the innate immune contributors that commence the pathogenesis (Jiang et al., 2022). PLT, a type of abundant blood cell, plays a central role in inflammation, immunology, hemostasis, and thrombosis (Eto & Kunishima, 2016). PLT becomes hyperactivated and produce a large number of platelet granules (Harifi & Sibilia, 2016), the most abundant of which are alpha granules containing a variety of cytokines, chemokines, and growth factors (Vieira-de-Abreu et al., 2012), which promote the overproliferation of fibroblast-like synoviocytes (FLS) to form vascular, tumor-like structures (Bartok & Firestein, 2010). These factors contribute to the formation of pannus, a tumor-like structure that penetrates bone and cartilage. The activated FLS secrete matrix metalloproteinases (MMPs), which degrade cartilage and bone, resulting in joint destruction (Bartok & Firestein, 2010). PLTs not only generate pro-inflammatory particles in peripheral blood and synovial fluid, but also have antigen-presenting properties, which can increase the immune response of T cells (Olumuyiwa-Akeredolu et al., 2019).

The infiltration of activated LYM and plasma cells was observed in the inflammatory synovial tissue of RA patients (Guo et al., 2018; Aletaha & Smolen, 2018), demonstrating the vital role of LYM in the pathogenesis of RA. Meanwhile, a prior study indicated that patients with active RA had reduced LYM counts (Symmons et al., 1989). Continuous migration and infiltration of peripheral blood LYM into the synovium, or  lymphocyte apoptosis, contributes to peripheral lymphopenia in patients with RA (Wehr et al., 2019; Weyand, Wu & Goronzy, 2020). Additionally, the high content of early apoptosis markers in LYM may also contribute to LYM depletion (Uslu et al., 2015; Du et al., 2017). In addition to a decrease in quantity, functional changes in LYM were also detected in RA, including impaired regulation of proliferation and differentiation, as well as an excessive inflammatory response (Ponchel et al., 2002; Szabó-Taylor et al., 2012). Under inflammatory conditions, activated B and T cells can promote osteoclast activation (Weitzmann, 2017), resulting in bone erosion (McInnes & Schett, 2011). In summary, PLT and LYM are involved in the pathogenesis and progression of RA. Therefore, the comprehensive index PLR is more responsive to the immune-inflammatory response of RA and provides more comprehensive and accurate information to help us determine the disease’s activity and better control its condition.

In comparison to previous reports, this study had some advantages. First of all, we employed a generalized linear model to estimate PLR’s association with DAS28-ESR and DAS28-CRP. We further applied a GAM to demonstrate the nonlinear connection between these factors, in order to describe the relationship between them more deeply. Secondly, we rigorously addressed potential confounding through multivariable adjustment across several models, which strengthens the internal validity of our observed associations. Third, when PLR was <254, for every 50-unit elevation in PLR, DAS28-ESR and DAS28-CRP increased by 0.126 or 0.145. The clinical significance of the present discovery is that only when PLR is lower than a certain threshold, PLR and DAS28-ESR and DAS28-CRP are positively correlated, but the mechanism needs to be further explored.

However, this study had limitations. Firstly, it is important to note that this study employed a cross-sectional observational design, which inherently limits our ability to establish direct causal relationships between exposures and outcomes. Therefore, further prospective studies with large samples are warranted. Secondly, our study included ERA cases who were hospitalized in our hospital, which does not completely represent all patients in China. Thirdly, the mechanistic underpinnings of the observed nonlinear association between PLR and DAS28 remain unexplored in this study. While plausible immunological and inflammatory pathways may mediate this relationship, the current investigation did not delve into these potential mechanisms. Fourth, our supplementary analysis indicated that the strength of correlation between PLR and the composite DAS28-CRP was not markedly greater than that between PLR and CRP alone. This suggests that PLR, as a marker of systemic inflammation, shares substantial variance with CRP in this context. Its potential independent utility for disease stratification beyond established acute-phase reactants may therefore be context-dependent and requires further delineation. Fifth, although we found that the PLR and DAS28-ESR and DAS28-CRP exhibit nonlinear characteristics with an obvious saturation threshold effect, this threshold is currently a purely data-driven result and requires external verification. Sixth, this study did not incorporate objective imaging data, such as joint ultrasound, and therefore could not directly link PLR with imaging-proven inflammation. However, the identified nonlinear relationship and the specific inflection point provide a critical entry point for subsequent research: future studies may validate whether specific PLR ranges correspond to ultrasonographic active synovitis or possess potential for predicting treatment response. Seventh, our core time variable was the symptom-to-admission interval (SAI), which reflects the cumulative symptomatic burden prior to the index hospitalization, regardless of whether it was a first or subsequent admission. Consequently, while our findings demonstrate an association between this cumulative symptom duration and inflammatory markers, they do not specifically inform on delays in the initial diagnosis of RA.

## Conclusion

A positive association was detected between PLR and DAS28-ESR and DAS28-CRP in ERA cases in China. Furthermore, the observed correlation exhibited nonlinear characteristics with a distinct saturation threshold effect. PLR was positively linked to DAS28-ESR and DAS28-CRP with PLR < 254.

## Supplemental Information

10.7717/peerj.21004/supp-1 Supplemental Information 1 Raw data

10.7717/peerj.21004/supp-2 Supplemental Information 2 Correlation of the platelet-to-lymphocyte ratio (PLR) with disease activity indicators in early rheumatoid arthritis (ERA) Abbreviations: PLR, platelet-to-lymphocyte ratio. Statistical note: All correlations were calculated using Pearson’s correlation coefficient. All P-values are less than 0.001.

10.7717/peerj.21004/supp-3 Supplemental Information 3 STROBE Statement Checklist of items that should be included in reports of observational studies

## Additional Information and Declarations

### Data Availability

The following information was supplied regarding data availability: The raw data are available in the Supplemental File.
