# Supplementary material for: A retrospective investigation of the platelet-to-lymphocyte ratio as a potential indicator in early rheumatoid arthritis
Source: PeerJ. 2026 Mar 31;14:e21004. doi: 10.7717/peerj.21004 (PMC13048224; doi:10.7717/peerj.21004)
Supplement: Supplemental Information 2 — Abbreviations: PLR, platelet-to-lymphocyte ratio. Statistical note: All correlations were calculated using Pearson’s correlation coefficient. All P-values are less than 0.001. [file peerj-14-21004-s002.docx]

**Table S1.** Correlation of the platelet-to-lymphocyte ratio (PLR) with disease activity indicators in early rheumatoid arthritis (ERA).

| Clinical Variable | Correlation with PLR (r) | P-value |
| --- | --- | --- |
| Erythrocyte sedimentation rate (ESR) | 0.420 | < 0.001 |
| C-reactive protein (CRP) | 0.420 | < 0.001 |
| Disease Activity Score in 28 joints using ESR (DAS28-ESR) | 0.396 | < 0.001 |
| Disease Activity Score in 28 joints using CRP (DAS28-CRP) | 0.405 | < 0.001 |

**Abbreviations:** PLR, platelet-to-lymphocyte ratio.

**Statistical note:** All correlations were calculated using Pearson’s correlation coefficient. All P-values are less than 0.001.
